# ‘Biomarking’ infection during continuous renal replacement therapy: still relevant?

**DOI:** 10.1186/s13054-015-0948-z

**Published:** 2015-05-21

**Authors:** Patrick M Honore, Rita Jacobs, Inne Hendrickx, Elisabeth De Waele, Viola Van Gorp, Herbert D Spapen

**Affiliations:** ICU Department, Universitair Ziekenhuis Brussel, Vrije Universiteit Brussel University, 101, Laarbeeklaan, 1090 Jette, Brussels, Belgium

We greatly appreciated the recent research article by Park and colleagues in *Critical Care* showing that procalcitonin (PCT) is not superior to C-reactive protein (CRP) as a marker for infection in renal impairment [1]. Additionally, we would like to comment on the validity of CRP and PCT measurement during continuous renal replacement therapy (CRRT). Both acute-phase proteins are indeed effectively cleared by CRRT. CRP is predominantly present as a monomer (mCRP) in the blood [2] and is removed by all forms of CRRT because its molecular weight (22-25 kDa) lies below the cutoff permeability limits of all classic dialysis membranes [3]. Most of the PCT mass is easily eliminated by convection [4]. However, although mCRP and PCT are adequately filtered, substantial amounts of both are adsorbed on the dialysis membrane [3, 4]. Therein lies a clinically relevant but poorly recognized problem! In fact, highly adsorptive dialysis membranes are increasingly applied to CRRT in many intensive care units worldwide. The use of such membranes will inherently accentuate mCRP and PCT removal. As a result, plasma levels of both biomarkers risk becoming falsely low during CRRT and thereby losing all potential to help clinicians diagnose or evaluate infection. The recently introduced sepsis biomarkers brain natriuretic peptide and N-terminal pro-brain natriuretic peptide have been proclaimed to be superior to CRP and PCT, but whether they perform better under CRRT is doubtful because their molecular weights (3.5 and 8.5 kDa, respectively) already predict highly effective clearance by both high- and low-flux membranes [5].

## Abbreviations

CRP: C-reactive protein
CRRT: Continuous renal replacement therapy
mCRP: Monomeric C-reactive protein
PCT: Procalcitonin
